# SiamOT: An Improved Siamese Network with Online Training for Visual Tracking

**DOI:** 10.3390/s22176597

**Published:** 2022-09-01

**Authors:** Xiaomei Gong, Yuxin Zhou, Yi Zhang

**Affiliations:** Department of Computer Science, Sichuan University, Chengdu 610017, China

**Keywords:** visual tracking, Siamese networks, online training

## Abstract

As a prevailing solution for visual tracking, Siamese networks manifest high performance via convolution neural networks and weight-sharing schemes. Most existing Siamese networks have adopted various offline training strategies to realize precise tracking by comparing the extracted target features with template features. However, their performances may degrade when dealing with unknown targets. The tracker is unable to learn background information through offline training, and it is susceptible to background interference, which finally leads to tracking failure. In this paper, we propose a twin-branch architecture (dubbed SiamOT) to mitigate the above problem in existing Siamese networks, wherein one branch is a classical Siamese network, and the other branch is an online training branch. Especially, the proposed online branch utilizes feature fusion and attention mechanism, which is able to capture and update both the target and the background information so as to refine the description of the target. Extensive experiments have been carried out on three mainstream benchmarks, along with an ablation study, to validate the effectiveness of SiamOT. It turns out that SiamOT achieves superior performance with stronger target discrimination abilities.

## 1. Introduction

Visual tracking is one of the most fundamental and active topics in the computer vision domain. Given the initial state (including the position and size of the bounding box) of an object in the first frame with virtually no prior knowledge, the tracker needs to continuously track the state of the object in the subsequent frames. In early years, template-based methods and correlation filters were the mainstream methods. For instance, the MOSSE [1] filter was a pioneering work in visual tracking using a correlation filter. In the next few years, with the growing popularity of deep learning, Siamese-based trackers sprung up with appealing performances. Albeit effective, they are facing two problems: First of all, most of them adopted offline training schemes, which mainly focused on the target features but overlook the background characteristics. The background information is also of vital importance to tracking tasks, especially the target surroundings. Secondly, an offline training scheme trains the network using image pairs, which could neither adapt to target changes nor recognize the unknown targets (those do not appear in the training sets). To realize end-to-end training, DiMP [2] announced a dedicated optimization and loss function, which fully exploited both target and background appearance information to predict target model within a few iterations. GradNet [3] is a novel gradient-guided network that updated the template in the Siamese network through feed-forward and backward operations. However, its shallow network did not well resolve the problem of background changes, which resulted in low tracking performance.

To remedy the above defects of Siamese networks, we propose integrating online training into Siamese networks in this paper. Compared with traditional Siamese networks, the online training branch could easily recognize the unknown targets, which weakens the influence of other disturbing factors. Combining the abundant target features extracted by the Siamese network with the online learned information would undoubtedly improve the tracking performance. In addition, we also employ feature fusion and attention mechanisms in our proposed online branch to make our tracker focus on more important target features.

**Figure 1:**
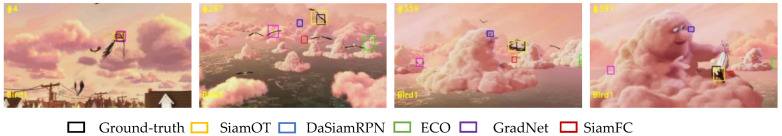
Comparative results among different trackers.

A sequence of tracking results is illustrated in Figure 1 between SiamOT and other popular methods (DaSiamRPN [4], ECO [5], GradNet [3], and SiamFC [6]). The black boxes are ground truth, and are manually annotated in each frame. The yellow boxes indicate our tracking results. Initially, all trackers target the wild goose. However, starting from the second frame, all other trackers either drift to other, similar geese, or point to the cloud clusters; only SiamOT always keeps track of the original goose.

In a nutshell, the main contributions to this paper can be summarized as follows:

1. We develop an online training module for a Siamese-based tracker, which aims to capture and update both the target and background changes. It turns out that the proposed module improves the target recognition ability of the tracker.
2. We investigate several feature fusion methods and propose a weighted fusion scheme, which integrates both deep and shallow features extracted by different layers of the backbone.
3. We devise an improved attention module and insert it into our tracking framework to make the tracker pay attention to more important channels so as to improve tracking efficiency.
4. Extensive experiments have been conducted on three mainstream benchmarks, along with ablation studies, to verify our design. SiamOT exhibits favorable performances.

The rest of the paper is organized as follows: Related works are discussed in Section 2. The architecture of our network is described in Section 3. The experimental results with ablation studies are shown in Section 4 with thorough analysis. A final conclusion is drawn in Section 6. 

## 2. Related Works

Normally, a generic tracking problem is decomposed into two steps, namely target classification and state estimation. The former step detects the target of interest in each frame, while the latter step regresses the properties of the target in the form of a bounding box (including central position and box size) that best describes the target in the current frame. Then, the tracker can associate the target across frames to form a trajectory. In view of the essence of tracking problems, the Siamese network became a suitable solution for visual tracking due to its two-branch structure, in which one branch is committed to classification and the other branch is used for regression. As a result, Siamese networks began to predominate the visual tracking field with astonishing advancement.

### 2.1. Siamese Networks

Considering the strong feature representation abilities of convolution neural networks (CNNs), SiamFC [6] applied the Siamese network to visual tracking for the first time using an offline training scheme. It generated the target features for both the first and the current frames based on Siamese structure, calculated their correlation to yield a response map, and finally located the target based on the position of its peak value on the response map. SPM-Tracker [7] elucidated a series–parallel matching algorithm to strengthen the robustness of the tracker on the basis of SiamFC. To improve the localization accuracy, SiamRPN [8] advocated a two-branch architecture, where one branch was responsible for classification (to distinguish target from the background) and the other branch was dedicated to a regression task (to estimate the position of target bounding box). In particular, SiamRPN [9] introduced the Region Proposal Network (RPN) for regression tasks. SiamMask [10] appended detection head to SiamRPN to create semantic segmentation results for the target. Zhu et al. [4] pointed out that the major drawback of current Siamese networks is the imbalanced distribution of training data, making the learned features less discriminative. They designed a distractor-aware module to perform incremental learning so as to facilitate target classification. Inspired by SiamRPN, Fan et al. [11] elaborated a multi-stage visual tracking framework to improve tracking accuracy and robustness, which was comprised of a sequence of RPNs cascading from high-level to low-level layers in a Siamese network. 

SiamRPN++ [12] was an improved version of SiamRPN, which analyzed the reason why deeper networks presented worse results than shallow networks. It then adopted a revised ResNet-50 as the backbone by replacing part of the convolution blocks with dilated convolutions. It also utilized anchor-based mechanism for object detection. However, an anchor-based mechanism needs prior knowledge about the ratio of the bounding box. Therefore, the tracker could not locate a newly appeared target that was not consistent with the prior knowledge. Zhang et al. [13] replaced the original AlexNet [14] backbone with a deeper and wider convolution network to improve robustness and accuracy. It also designed new cropping-inside residual units (CIR) to eliminate the negative impact of network padding. To overcome the problem of data drift, Tang et al. [15] provided a template adjustment module to adapt to the target appearance variation in a long-term sequence.

To overcome the inherent limitations of anchor-based mechanisms coping with unknown targets. SiamPCF [16] located targets in an adaptive manner. Instead of using the bounding box directly, SiamPCF transformed the points on the target to a bounding box to describe the target more accurately. SiamBAN [17] was an anchor-free based tracker, which announced an elliptical-shaped labeling method to locate the target. AF2S [18] replaced the anchor-based RPN with a two-stage tracker based on SiamFC. Zhang et al. [19] introduced a feature alignment module to learn object-aware features within the predicted bounding boxes. Peng et al. [20] regressed the attributes of the bounding box without additional classification or regional proposals, and tuned the stride and receptive field for the network. Compared with the abovementioned methods, Held et al. [21] realized a high-speed tracking network (100 fps) via simple feed-forward network without online training. Despite its high speed, its accuracy was less satisfactory, since it derived the target position simply based on previous frame.

The core idea of Siamese networks is the calculation of similarity between the template patch and the search patch. However, most existing Siamese networks adopted offline training schemes, which lacked the learning and updating of target changes during tracking. Moreover, some Siamese networks excel at identifying the target from the background, but they are easily disturbed by similar targets or other interferences.

### 2.2. Template Update Algorithms for Visual Tracking

ATOM [22] was a novel and high-speed tracker, which incorporated dedicated target estimation and classification components. It computed the overlapped region (IoU: intersection over union) between the predicted and ground truth boxes and maximized the IoU to locate the target using a gradient descent method. UpdateNet [23] replaced the handcrafted update function with a lightweight auto-learning module. An optimal template for the next frame was estimated based on a combination of the initial template, the accumulated template, and the current frame. GradNet [3] was a novel gradient-guided network used to solve the over-fitting problem, which exploited the discriminative information in gradients and updated the template through feed-forward and backward operations. However, it suffered the same limitations as other methods of short memory of the past frames. For this reason, LSTM modules were implanted to store historical information [24,25,26], but they were computationally heavy. MDNet [27] encompassed shared layers and multiple branches of domain-specific layers to identify the target in each domain. However, it failed to meet real-time demand. MBMD [28] developed a long-term tracking framework based on a deep regression and verification module to generate a series of candidates and to predict their similarity scores based on object-aware feature fusion and RPN. Yao et al. [29] investigated the joint learning of deep representation and model adaptation, and truncated the alternating direction method of multipliers (ADMM). Choi et al. [30] put forth a template selection strategy using deep reinforcement learning methods.

A variety of template update strategies have been developed by the above-mentioned methods, with proven improvements. Nevertheless, simple update mechanisms could not handle unpredictable target changes or complex scenes, while more complicated algorithms would inevitably increase the computational cost.

### 2.3. Emerging Methods in Visual Tracking

Recently, meta-learning has been widely applied to visual tracking. Park et al. [31] developed an offline meta-learning-based method to adjust the initial deep networks used in online adaptation-based tracking. On the other hand, motivated by the great success of transformer in natural language processing (NLP), researchers attempted to migrate it to a computer vision domain to implement downstream vision tasks. Aiming to solve the insufficient global information and weak feature extraction ability of existing trackers, Hu et al. [32] advised using a novel tracking architecture that combined feature enhancement and template update. Yan et al. [33] came up with an encoder–decoder transformer to estimate the positions of target corners. However, some of the transformer architectures relied on high-end equipment (e.g., 8 × V100 cards are required to run [33]). Except for a few commercial institutions, other units cannot afford such an expensive platform to replicate the results.

Considering that Siamese networks are still one of the dominant methods in visual tracking, in this paper, we mainly focus on improving the tracking performance of Siamese-based networks using an online training scheme. Especially, we implant an online training branch to enhance the adaptation ability of target changes. Our network structure will be described in detail in Section 3 below.

## 3. Siamese Networks with Online Training Module

**Figure 2:**
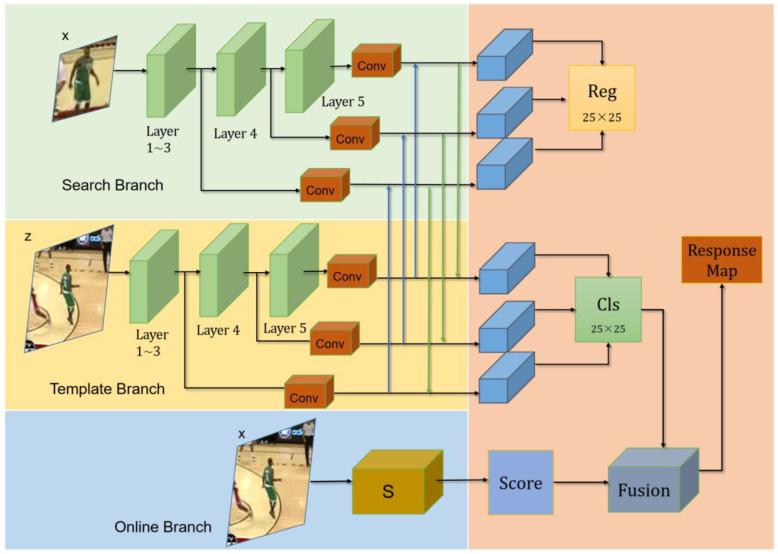
The overall structure of SiamOT.

The overall structure of SiamOT is illustrated in Figure 2 below. We use ResNet-50 as our backbone. The yellow and the green blocks represent classification (cls) and regression (reg) branches, respectively, and the blue block denotes the online training (OT) branch.

### 3.1. Problem Formulation

For a typical Siamese network, the score maps of classification and regression branches are written as:

(1) $$\{\begin{array}{c} s{\left(z,x\right)}^{cls}=\sum_{i=3}^{5}\alpha_{i}(\varphi_{i}(z)*\varphi_{i}(x))\\ s{\left(z,x\right)}^{reg}=\sum_{i=3}^{5}\beta_{i}(\varphi_{i}(z)*\varphi_{i}(x)) \end{array}.$$

Here, *z* and *x* represent the cropped patches in the first frame and the current frame. *α_i_* and *β_i_* are the weights for classification and regression branches, respectively. *φ_i_* is the feature extractor. * refers to the convolution operation.

The purpose of the proposed online training module is to capture and update the target changes. The score map of the online training module is calculated as:

(2) $$s^{online}=s_{C}\left(Att(f(x));\theta \right)$$

Here, *f*(*x*) indicates the extracted feature of *x*. *Att* represents the attention mechanism. *θ* is the set of weights of online training module; *s_C_* refers to an online training function. As shown in Figure 2, *s^online^* is fused with the output of classification branch (*s*(*z*,*x*)*^cls^*) to better distinguish the target from the background. The details of the entire network will be described in Section 3.2 below.

### 3.2. Online Training Module (OT)

**Figure 3:**
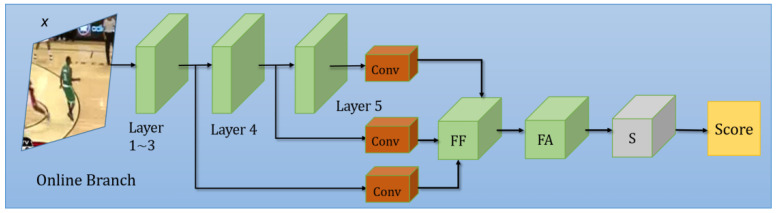
The online training (OT) branch of our network.

The online training module is illustrated in Figure 3. It consists of a feature fusion sub-module (FF), a feature attention sub-module (FA), and a training sub-module (*s*). The features from the third, the fourth, and the fifth layer of the backbone are first processed by convolution blocks, and are then fused by FF. Then, the important features are filtered by FA, and are finally fed to *s* to yield the final score map *s^online^*. During training, *x* is augmented by our OT branch to create a training set, and the weights of *s* are also updated at the same time. The OT branch is explained in detail in Section 3.2.4. 

#### 3.2.1. Feature Fusion (FF)

The feature that is fed to the training sub-module is critical for online training. Some of the previous methods (published after SiamRPN++) employed ResNet-50 as the backbone in a weight-sharing manner. Other methods [20] directly utilized the deep features from the fifth layer. Normally, the deep features contain more semantic information, and the shallow features carry spatial details, which are all beneficial for tracking. Considering this, we investigate several feature fusion methods, including simple addition (3), multiply (4), and concatenation (5) as follows:

(3) $$f{\left(x\right)}_{add}=\sum_{i=3}^{5}\varphi_{i}(x)$$

(4) $$f{\left(x\right)}_{Multiply}=\prod_{i=3}^{5}\varphi_{i}(x)$$

(5) $$f{\left(x\right)}_{Concat}=concat(\varphi_{3}(x),\varphi_{4}(x),\varphi_{5}(x)).$$

Here, *φ*_3_(*x*), *φ*_4_(*x*) and *φ*_5_(*x*) denote the extracted feature after the third, the fourth, and the fifth layer of backbone (shown in Figure 3 above). *concat* denotes the concatenation operation. 

However, the above three fusion methods yield less satisfactory results. Thus, we try the following weighted fusion method:

(6) $$f{\left(x\right)}_{wf}=\sum_{i=3}^{5}\alpha_{i}\varphi_{i}(x)$$

where *α_i_* is the weight for each extracted feature. It turns out that the weighted fusion method produces the best results. A comparison of the above methods (3)–(6) is provided in the ablation study in Section 5.1.1.

#### 3.2.2. Attention Mechanism

**Figure 4:**
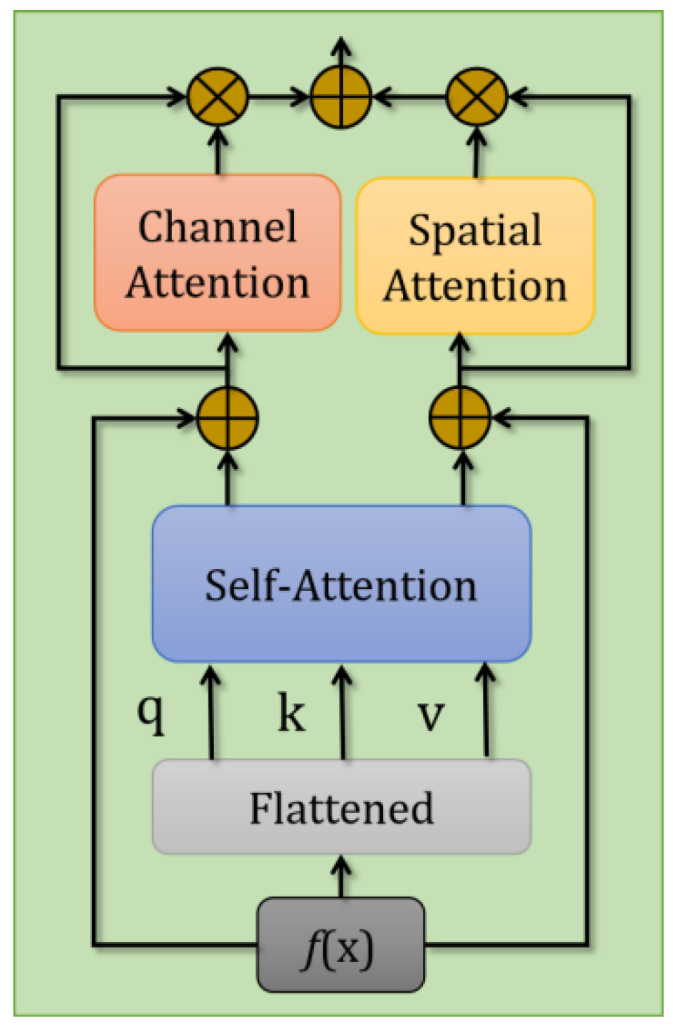
The architecture of the proposed attention module in SiamOT.

SiamRPN++ [12] visualizes the features of different channels to prove that only part of the channels contribute to the final score map. Inspired by [12,34], we add an attention module into our tracking framework to focus more on important channels. Our proposed attention assisted network architecture is shown in Figure 4, in which input *f*(*x*) is a fused feature calculated by (6) above. Instead of directly using the structure in [34], we insert channel attention and spatial attention modules into the original structure to calculate the corresponding attentions in the feature map so as to pay more attention to the important channel and spatial location. 

In order to strengthen the connection between each 1 × 1 × 256 feature of *f*(*x*), the fused feature is flattened into *q*, *k*, *v* (query, key, value) components and are enhanced as:

(7) $$Att_{s}=softmax(qk^{T}\delta )v+f(x)$$

Here, *δ* is a weight with the same dimension as *f*(*x*). *Att_s_* is the enhanced feature via attention mechanism (with dimension channel × *w* × *h*). First of all, we flatten *f*(*x*) (*h* × *w* × 256) into *hw* × 256. Meanwhile, *q* is *hw* × 256 and *k^T^* is 256 × *hw*, so *qk^T^* calculates the relation between each 1 × 1 × 256 dimensional feature and the remaining *h* × *w* − 1 features via inner product multiplication. Then, *softmax* function is used to normalize the relation of each row. Finally, the normalized results are added by the original *f*(*x*) to yield rich target features (such as a residual structure).

The results of the self-attention module are fed into channel and spatial attention modules, respectively, for further feature enhancement in channel and spatial dimensions. To be more specific, the following equation is devised for the channel attention module:

(8) $$W_{channel}=Sigmoid(Relu(Avgpool(Att_{s})w_{c1})w_{c2})$$

Here, *Avgpool* refers to average pooling operation; *w_c_*_1_ and *w_c_*_2_ are the corresponding weights. Firstly, we perform an *Avgpool* on the enhanced features *Att_s_*, followed by *Relu* and *Sigmoid* activation functions to produce the final weights for the channel attention module *W_channel_*.

Similarly, for spatial attention module, we generate the weights as:

(9) $$W_{spatial}=Sigmoid(Att_{m})$$

Here, *Att_m_* (with dimension 1 × *w* × *h*) is the mean value of *Att_s_* along the channel dimension (after the mean operation, the channel size becomes one). Then, the features processed by spatial and channel attention modules are expressed as:

(10) $$Att_{spatial}=Att_{s}⊗W_{spatial}$$

(11) $$Att_{channel}=Att_{s}⊗W_{channel}$$

⊗ denotes element-wise multiplication. Finally, a fusion operation is performed as follows:

(12) $$Att=Att_{channel}⊕Att_{spatial}$$

⊕ denotes element-wise addition.

#### 3.2.3. Online Learning Function

Danelljan et al. [2] declared that classical Siamese networks could only learn target features from image pairs through offline training. In contrast, online trained networks could learn both the target and the background information. In this light, we append the online training branch to our architecture. After a series of feature fusion and augmentation operations, we apply a learning function (*s* in Figure 3) to calculate the score map according to (2), so as to distinguish target from other distractions. The loss of the proposed learning function is calculated as:

(13) $$L\left(s\right)={\left\|s^{online}-y_{g}\right\|}^{2}$$

Here, *L*(*s*) is the loss value of online training, *s^online^* represents the score map in (2), and *y_g_* is the ground truth value. We adopt Gaussian distribution to model the target position, using *y_g_* as the center. 

Compared with offline training, the online training scheme captures richer target features. However, it has sparse training samples; thus, we utilize historical frames as the training sample with data augmentation techniques. 

#### 3.2.4. The Fusion of Siamese Network with Online Training Branch

As described earlier, Siamese networks and online training schemes have their own advantages. For instance, the former could be trained by a large amount of image pairs to ensure high recognition accuracy. The latter updates the target changes. Based on this, we combine them together to compute a more accurate score map. The most straightforward way to combine them is by simply adding them together:

(14) $$s{\left(z,x\right)}^{final}=s{\left(z,x\right)}^{cls}+s^{online}$$

However, the result is less satisfactory. Instead, it turns out that a weighted fusion demonstrates much better results:

(15) $$s{\left(z,x\right)}^{final}=(1-\eta )\times s{\left(z,x\right)}^{cls}+\eta \times s^{online}$$

Here, *η* is set to 0.8. An ablation study is conducted in Section 5.1.3 to verify this empirical value.

#### 3.2.5. Online Tracking Algorithm Flow

In this section, we will explain the processing flow of our network. Given the first frame, the input *z* is generated based on the target position. Then, a data augmentation method [35] is applied to the first frame to yield the training set *T*, which is used for initial online training. During tracking, we send *x* (generated by the test frame) to the two branches to produce two score maps, which are fused to obtain the target center. Finally, the target bounding box is created by the regression branch. The entire online tracking flow is written in Algorithm 1. The parameters involved in online training and template updating will be discussed in Section 4 below.
**Algorithm 1** Flow of Online Tracking**Input:** The 1st frame of the video sequence *I_0_*; The initial target bounding box *b*_0_ **Output:** The current target bounding box *b_t_* 1: Create cropped patch *z* based on *b*_0_ from first frame *I*_0_. Create the training set *T* based on *I*_0_, which is used to online train online the network *s*. 2: **for**
*t* = 1, …, *N*
**do** 3:  Create search patch *x* based on *I_t_* 4:  Obtain *s^cls^*(*z*,*x*) using Equation (1) 5:  Obtain *f*(*x*) using Equation (6) 6:  Obtain *Att_s_* using Equation (7) 7:  Obtain *Att* on the basis of *Att_s_* using Equations (8)–(12) 8:  Calculate score map *s^online^* using Equation (2) 9:  Calculate *s^final^* using Equation (14) 10:   Obtain the target bounding box in current frame based on *s^final^* and *s^reg^*(*z*,*x*) 11:   **if** (*t* mod *C*) = 0 **then** 12:    Obtain the cropped patch *φ*(*z*) based on *b_t_* 13:    Replace the template of Siamese network by current frame *φ*(*z*_0_) = *φ*(*z*) 14:   **end if** 15: **end for**

## 4. Experimental Details

The following experiments are built on the Pytoch framework with Python. The detailed experimental configurations are as follows (Table 1):

The initial weights of our network are set by referring to SiamBAN. Since we adopt online training scheme, the traditional gradient update algorithms are not involved and discussed here. We employ translation, flip, blurring, and rotation for data augmentation to produce 30 training samples with dimensions 31 × 31 × 256. Then, the Gaussian score map is generated as the ground-truth label, in which the real target center is treated as its center.

After the generation of training samples and ground-truth values, we perform 10 iterations of gradient descents to initialize the optimizer. After initialization, all training samples are sent to attention module for another 60 iterations of training. In the following tracking process, feature fusion and enhancement are carried out for each input patch *x*. *s^online^* is calculated through Figure 3 to fuse the final score map via (15). As suggested by [5,22,34] The update interval (C in Table 1) is set to five.

## 5. Experiment

In this section, we conduct comparative experiments on three popular datasets, including OTB100 [36], LaSOT [37] and NFS [38] to evaluate the performances of our tracker. 

**OTB100:**

OTB100 is a popular benchmark for performance evaluation, including 100 short videos. The evaluation metrics are success rate and precision. 

In this experiment, we compare our tracker with classical Siamese networks (e.g., SiamFC, SiamRPN++ and SiamBAN), online training methods (e.g., ATOM, DiMP, and MDNet) and some offline training methods. As shown in Table 2, our tracker ranks second in both success rate and precision. 

**Figure 5:**
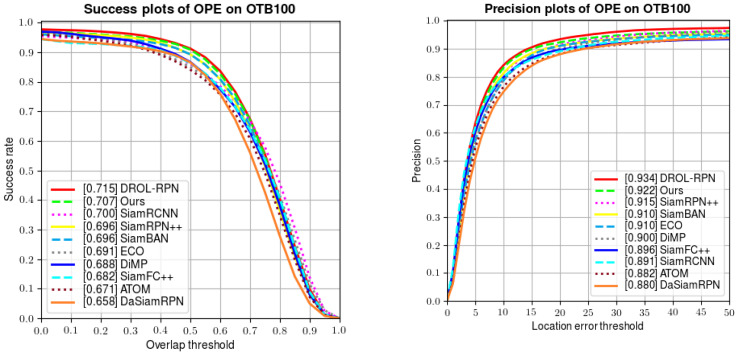
Comparative results of success rates and precision on OTB100.

The evaluation metrics Succ and Prec. characterize the areas under the curves in Figure 5 below. Compared with the classical Siamese network SiamFC, we improve the success rate and precision by 20.4% and 27.7%, respectively. Compared with the recently published DSiamRPN, we also improve the two indexes by 7.4% and 4.8%, respectively. Meanwhile, we also attain some performance gain over excellent tracker SiamRPN++. Additionally, we surpass SiamRCNN [41] in success and precision by 1% and 4%. We only lag behind DROL-RPN [42]. However, we obtain better results than DROL-RPN for fast motion, low resolution, and large variation.

The above experiment on OTB100 validates the effectiveness of our tracker, which proves the effect of an online training branch in improving the performance of original Siamese networks. Although classical Siamese networks extract useful target features, they are limited by the size of training sets and cannot adapt to target changes. Under such circumstances, our proposed online training branch remedies the above shortcomings by adjusting the weights during the training process to learn rich target features, so as to ensure the robustness of tracking.

On the other hand, compared with online-training-based trackers (including MDNet, ATOM, and DiMP), we also exceed them in success rates and precisions by some margins. The results demonstrate the fact that the Siamese network and online training branch are individually effective and are also complementary in boosting the tracking performance.

**Figure 6:**
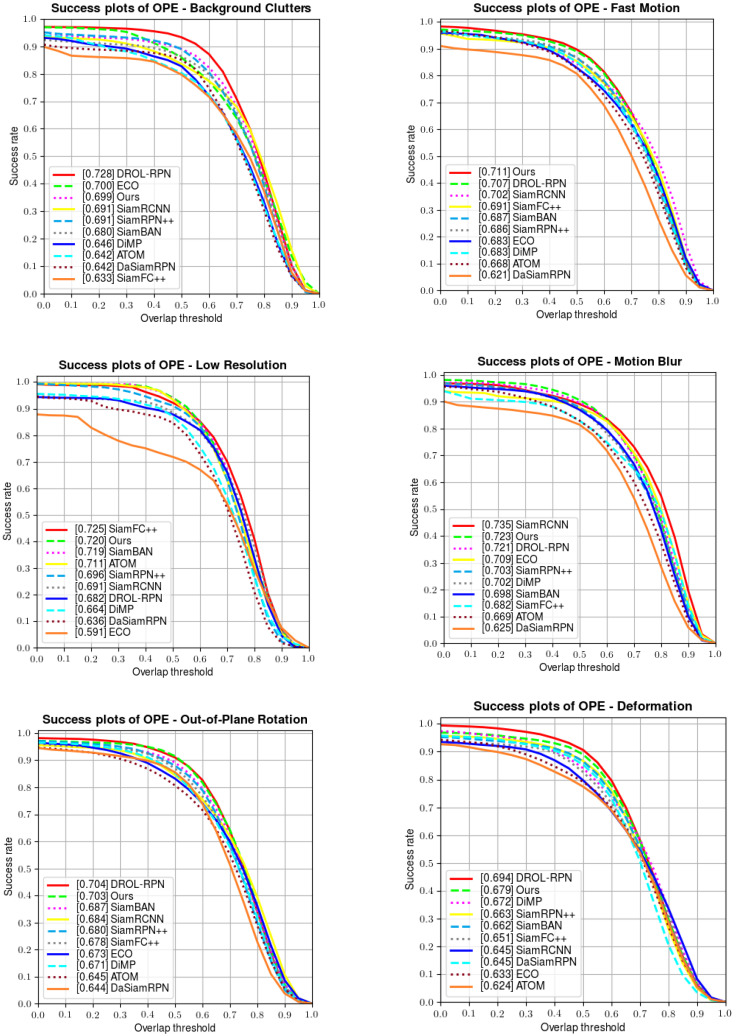

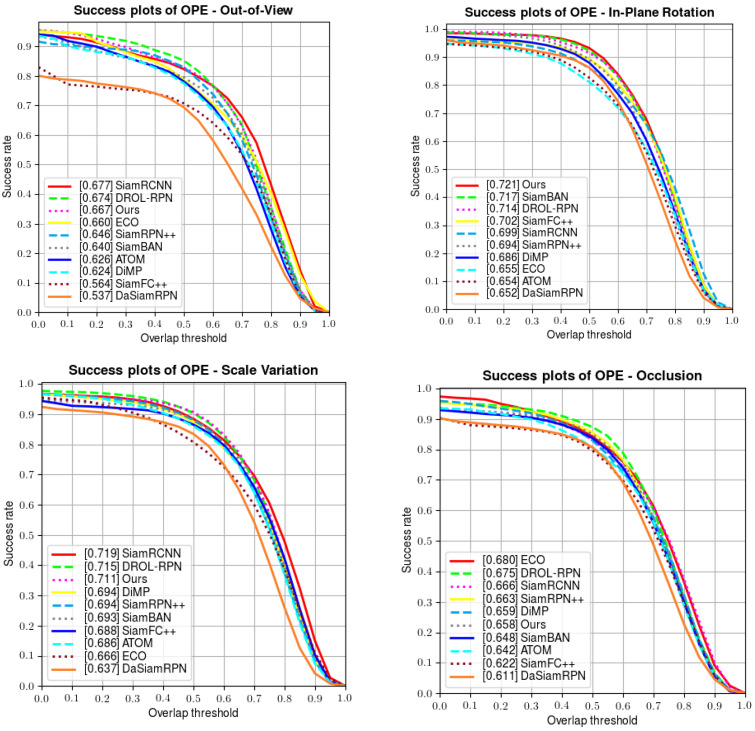
Comparisons of the attributes on OTB100.

Apart from the above results, OTB100 also provides several attributes to test the robustness of each tracker in dealing with unpredictable challenges (including illumination variation, fast motion, scale variation, etc.). A series of comparisons are made in Figure 6 below; our tracker manifests favorable performances when handling fast motion, deformation, rotation, out-of-view, illumination variation, and scale variation, and also achieves a competitive performance in solving background clutter and low-resolution situations.

**Figure 7:**
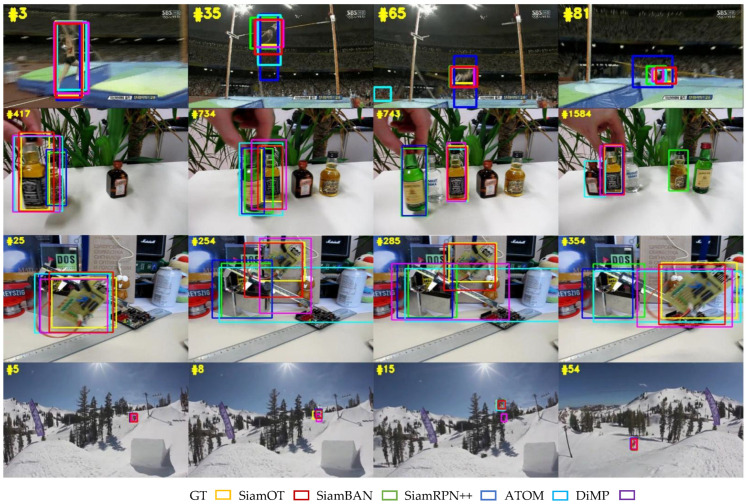
Qualitative comparison of our tracker with other popular trackers on 4 video sequences from OTB100.

A qualitative comparison between different trackers on OTB100 is illustrated in Figure 7 below. The first video (the first row) involves target deformation, the second video (the second row) contains multiple similar objects, the third video (the third row) shows a rotating object, and the last video (the right row) is an example of small object. In these four situations, our tracker exhibits strong robustness against other popular methods when coping with adverse situations.

**LaSOT:**

**Figure 8:**
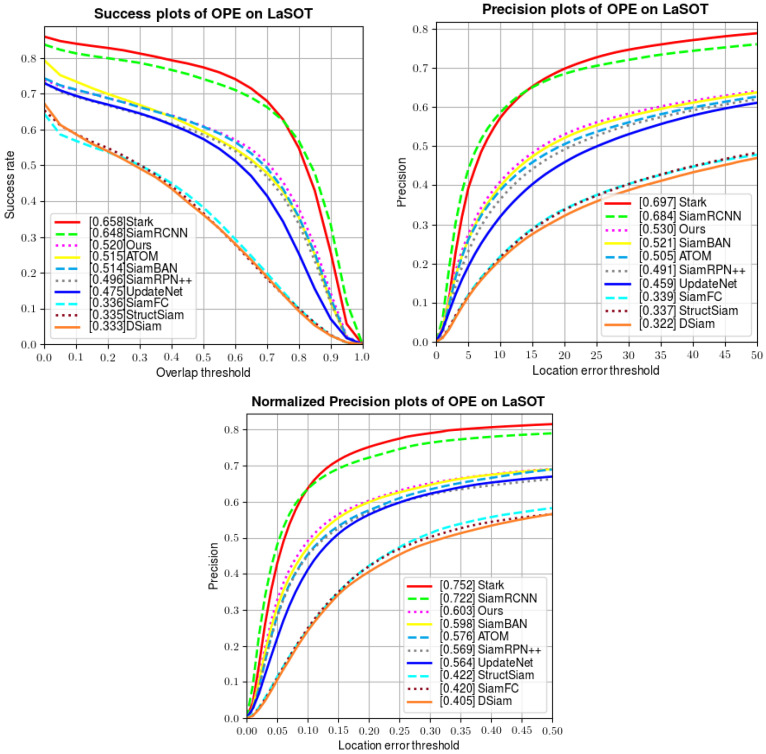
Comparison of tracking performance on LaSOT.

Unlike OTB100, which has only short videos, LaSOT is a huge and widely used long-term dataset including 1400 videos, wherein each of them has more than 2500 frames on average. It also includes challenging situations such as background clutter, fast motion, scale variation, etc. We evaluate our tracker using 280 sequences on the test set of LaSOT. A quantitative comparison is made in Figure 8, showing the success rate, precision, and normalized precision of several trackers. Compared with both popular offline-training-based trackers (SiamFC [6], SiamBAN [17], SiamRPN++ [12], StrcutSiam [43], DSiam [44], UpdateNet [23], SiamRCNN [41], and Stark [33]) and online-training-based trackers (ATOM [22]), our tracker wins third place in all three metrics. The results on LaSOT demonstrate that our tracker excels at tracking tasks in long-term videos.

Unlike the previous experiment on OTB100, in this experiment, we obviously lag behind Siam R-CNN and STARK. Our analysis of this result is as follows: the main innovation of Siam R-CNN is the combination of the re-detection module and the dynamic tracking algorithm. It measures the similarity between the region proposal and the template patch and updates the bounding box in a dynamic way, so that when the tracker loses the target, it regains the target in a short time. Similarly, STARK advised a score prediction head to update the template to ensure the robustness in the tracking process. In comparison, although our online module updates the template to capture target changes, it cannot re-detect the target when it disappears temporarily. We think an additional local search strategy would help with target re-detection.

**NFS:**

NFS contains 100 videos of real scenes captured by higher-speed cameras, in which we select NFS30 (videos with 30 fps) for testing. The evaluation metrics are similar to OTB100. The comparative results are listed in Table 3 above.

As expected, our tracker again ranks first in both success rate and precision on NFS among all the popular methods. Since the videos in NFS are all from real life (YouTube life videos), the results on NFS have high practical significance.

### 5.1. Ablation Study

In Section 3.2.1, we discussed multiple feature fusion schemes, including addition, concatenation, multiplication, and weighted fusion. In this section, we test and compare different fusion schemes and attention mechanisms along with key parameters to analyze the optimal configuration.

#### 5.1.1. Fusion Scheme

We investigate the following feature fusion schemes (expressed as Equations (3)–(6)) to extract rich semantic features, and search for the best strategy:

As shown in Table 4, the weighted fusion method (Equation (6)) has the highest success rate and precision. We thereby utilize weighted fusion to integrate the two branches.

#### 5.1.2. Attention Mechanism

In this section, we test different combinations of attention mechanisms on a NFS30 dataset to testify their effectiveness. As shown in Table 5, without any attention modules, the success rate is only 0.503. When different attention modules are added into the network, the success rate increases significantly. Finally, when we add self-attention, spatial attention, and channel attention modules, we achieve the highest success rate.

#### 5.1.3. The Weights in Fusion Process

In this section, we test different values of parameter *η*, which balances the weights between the Siamese network and the online training branch. We believe the target state in the online branch should be given higher weights than the classification branch (i.e., *η* > 0.5). To search for the optimal value of *η*, we list different success and precision results using different values of *η* (shown in Table 6). Fortunately, when *η* = 0.8, we obtain the highest success and precision values, which confirms our conjecture. Therefore, we select *η* = 0.8 in the weighted fusion process.

## 6. Conclusions

In this paper, we develop a Siamese-based tracking network integrated with an online training branch, where the core components include feature fusion and improved attention modules. Our proposed architecture not only extracts rich target features to ensure tracking accuracy and efficiency, but also proves that the classical Siamese network and the online training strategy could be organically combined and are complementary in tracking tasks. Extensive experiments have been conducted on three prevailing tracking benchmarks. The experimental results verify the effectiveness and advancement of our tracker compared with other popular methods. We hope our work will provide a reference for those who are studying Siamese networks for visual tracking. Meanwhile, we are now investigating the combination of convolution blocks and transformer structure, and plan to apply it to visual tracking in the near future.

## Figures and Tables

**Table 1:** Experimental environment.

Hardware	GPU	Nvidia RTX 3090
	CPU	Intel^®^ Core™ i7-9700 K CPU @ 3.60 GHz × 8
Software	OS	Ubuntu 20.04
	Framework	Pytorch 1.7.0
	Language	Python

**Table 2:** Comparative results on OTB100.

Trackers	SUCC.	PREC.
GradNet [3]	0.639	0.861
UpdateNet [23]	0.647	0.861
ATOM [22]	0.671	0.882
MDNet [27]	0.678	0.909
DSDCF [39]	0.667	0.784
DiMP [2]	0.688	0.900
ECO [5]	0.691	0.910
DaSiamRPN [4]	0.658	0.880
SiamFC [6]	0.587	0.722
SiamFC++ [40]	0.682	0.896
SiamBAN [17]	0.696	0.910
SiamRPN++ [12]	0.696	0.915
SiamRCNN [41]	0.700	0.891
DROL-RPN [42]	0.715	0.934
Ours	0.707	0.922

**Table 3:** Comparative results on NFS.

Trackers	SUCC.
UPDT [35]	0.537
MDNet [27]	0.422
C-COT [45]	0.488
ECO [5]	0.466
ATOM [22]	0.584
DiMP [2]	0.620
SiamBAN [17]	0.594
Ours	0.630

**Table 4:** Comparison of different feature fusion methods.

Fusion Strategy	SUCC.	PREC.
Addition	0.704	0.916
Concatenation	0.706	0.915
Multiplication	0.704	0.920
Weighted Fusion	0.707	0.922

**Table 5:** Comparison of results of different attention mechanisms on NFS.

Attention Mechanism	SUCC.
None	0.503
Self-Attention	0.565
Self-Attention + Spatial Attention	0.589
Self-Attention + Channel Attention	0.591
Self-Attention + Spatial Attention + Channel Attention	0.630

**Table 6:** The comparative results of success and precision using different weights.

*η*	SUCC.	PREC.
0.1	0.693	0.907
0.2	0.688	0.896
0.3	0.698	0.909
0.4	0.704	0.919
0.5	0.705	0.911
0.6	0.706	0.921
0.7	0.701	0.912
0.8	0.707	0.922
0.9	0.701	0.915
